# Reintroductions and genetic introgression from domestic pigs have shaped the genetic population structure of Northwest European wild boar

**DOI:** 10.1186/1471-2156-14-43

**Published:** 2013-05-20

**Authors:** Daniel J Goedbloed, Pim van Hooft, Hendrik-Jan Megens, Katharina Langenbeck, Walburga Lutz, Richard PMA Crooijmans, Sip E van Wieren, Ron C Ydenberg, Herbert HT Prins

**Affiliations:** 1 Resource Ecology Group, Wageningen UR, P.O. Box 47, Wageningen 6700AA, the Netherlands; 2 Animal Breeding and Genomics Centre, Wageningen UR, P.O. Box 338, Wageningen 6700AH, the Netherlands; 3 Wildlife Research Institute, Pützchens Chaussee 228, Bonn 53229, Germany

## Abstract

**Background:**

Population genetic studies focus on natural dispersal and isolation by landscape barriers as the main drivers of genetic population structure. However, anthropogenic factors such as reintroductions, translocations and wild x domestic hybridization may also have strong effects on genetic population structure. In this study we genotyped 351 Single Nucleotide Polymorphism markers evenly spread across the genome in 645 wild boar (*Sus scrofa*) from Northwest Europe to evaluate determinants of genetic population structure.

**Results:**

We show that wild boar genetic population structure is influenced by historical reintroductions and by genetic introgression from domestic pigs. Six genetically distinct and geographically coherent wild boar clusters were identified in the Netherlands and Western Germany. The Dutch Veluwe cluster is known to be reintroduced, and three adjacent Dutch and German clusters are suspected to be a result of reintroduction, based on clustering results, low levels of heterozygosity and relatively high genetic distances to nearby populations. Recent wild x domestic hybrids were found geographically widespread across clusters and at low frequencies (average 3.9%). The relationship between pairwise kinship coefficients and geographic distance showed male-biased dispersal at the population genetic level.

**Conclusions:**

Our results demonstrate that wildlife and landscape management by humans are shaping the genetic diversity of an iconic wildlife species. Historical reintroductions, translocation and recent restocking activities with farmed wild boar have all influenced wild boar genetic population structure. The current trend of wild boar population growth and range expansion has recently led to a number of contact zones between clusters, and further admixture between the different wild boar clusters is to be expected.

## Background

Most population genetic studies consider dispersal and isolation by landscape barriers to be the main drivers of genetic population structure [1]. However, human activities such as reintroductions, translocations and genetic introgression from domestic sources, may play an important role in certain study systems, in addition to natural dispersal and landscape patterns [2-4]. Such human activities, legal or not, are often poorly documented and their population genetic effects are mostly unknown. Molecular techniques provide increasingly powerful and affordable tools to evaluate anthropogenic influences on wildlife genetic population structure [5,6]. The use of Single Nucleotide Polymorphisms (SNPs) in particular is promising for the fields of population and conservation genetics [7,8].

Wild boar became extinct in large parts of Western Europe in the 19^th^ century [9]. The species was marginalized mainly by overhunting and deforestation associated with increased agricultural land use. Extinction in Britain had already occurred in the 13th century [10]. This massive decline in Western Europe was followed by an unknown number of mostly undocumented reintroductions in the late 19^th^ and early 20^th^ century. One such event is the commonly known but undocumented reintroduction of wild boar to the Veluwe, the forested centre of The Netherlands, which occurred in 1904 at the orders of Hendrik, Prince-Consort of Queen Wilhelmina of The Netherlands, for the purpose of hunting [11]. These animals are thought to stem from Northeast Germany and Czech Republic.

Conditions for wild boar steadily improved during the 20^th^ century due to hunting restrictions, reforestation, changes in agriculture and possibly climate change [12,13]. Starting from 1960, wild boar populations throughout Europe saw rapid growth and range expansion [14,15]. Wild boar (*Sus scrofa*) are adaptive and opportunistic omnivores as well as good dispersers, being able to travel distances up to 250 km [16] and fast breeders, with litter sizes of 4–7 once a year [9]. Dispersal is male-biased in this species [9,17]. European wild boar population structure at the continental scale is mainly shaped by post-glacial colonization patterns [18]. It is, however, unknown how the history of marginalization, reintroductions and recent population expansion has affected the genetic population structure at local or regional scales. In an area such as The Netherlands and Western Germany, one could expect high rates of gene flow.

Wild boar farming became popular in Europe in the second half of the 20^th ^century to provide for a demand in luxury meat. Hybridization between wild boar and domestic breeds is common practise on these farms to achieve increased reproduction and growth rates [19]. Such hybrids have been shown to be the source of the escaped wild boar population in England [20]. Introduction of wild boar originating from hybrid farmed stocks has also been shown in mainland Europe [21]. This has effectively led to genetic introgression from domestic pigs into local wild boar populations. Recent hybrids (until 5^th ^generation backcrosses with wild boar) as well as advanced generation hybrids (resulting from reproduction among hybrids across multiple generations) were identified. However, the spatial extent of domestic introgression and its effects on the population genetic structure of European wild boar have not been studied in detail.

From an evolutionary point of view, possible adverse effects of genetic introgression from a domestic or hybrid source include genetic adaptation to captivity and possibly outbreeding depression [22], while possible advantageous effects include hybrid vigour, increased growth rates and larger litter size. These evolutionary advantageous effects may be undesirable from a management perspective, as more rapidly reproducing wild boar can be difficult to control using normal population management practices and can then cause significant damage to agricultural crops [23]. Strikingly high litter sizes and strong differences in litter size between regions have indeed been observed in wild boar in Germany [24]. In addition to evolutionary effects, also population composition and structure can be affected by hybrid introductions and restocking practices [25].

In this study we used 351 SNP markers, genotyped for 645 wild boar, including 88 samples from a previous study [21], to assess the effects of historical marginalization, reintroductions and genetic introgression from domestic pigs on the population genetic structure of wild boar in The Netherlands and Western Germany.

## Methods

Blood or tissue samples were taken from a total of 645 wild boar in parts of The Netherlands, Western Germany and Luxembourg. This included 88 samples from a previous study [21], which were genotyped using the Illumina porcine SNP60 genotyping beadchip [26]. All samples were collected in the years 2008–2010 from animals identified in the field as wild boar. Sampling was performed on animals culled by wildlife managers for reasons of routine wildlife management or in the context of obligate disease monitoring programs. No animals were killed or inconvenienced for the purpose of this study. This study was approved a priori by Laboratory Animal Science officials in compliance with Dutch law.

DNA was extracted using the Qiagen PureGene (Blood) kit protocol. Samples were genotyped for 384 SNPs selected from the Illumina porcine SNP60 genotyping beadchip [26] from loci known to be polymorphic in wild boar in the study area, with proportional coverage of each chromosome and random selection within each chromosome. Of these 60k SNPs, 76% proved to be polymorphic in our wild boar dataset. Random selection within the autosomal and X chromosomes was performed to minimalize ascertainment bias. The only possible remaining ascertainment bias in our SNP set is derived from the ascertainment panel of the Illumina porcine SNP60 genotyping beadchip itself, and is considered to have no effect on the inference of wild boar population structure in the study area. Less than 0.0033% of the pairwise distances between the 351 randomly chosen SNPs were closer than 50,000 bp, which is considered to be the maximum range of physical linkage in wild boar [27]. Selected SNPs were genotyped on an Illumina GoldenGate bead array platform (BeadXpress, Illumina Inc.) in a 96 well, 384 SNP format [28]. Genotyping quality was assessed using GenomeStudio software (Illumina Inc.). Low genotyping quality or lack of differentiation between homozygote and heterozygote clusters lead to the removal of 33 SNPs. This left 351 non-coding SNPs for data analysis (Additional files 1 and 2), which is roughly equivalent in statistical power to 140 microsatellites [29,30].

Linkage Disequilibrium (LD) was analysed in plink v1.06[31] by calculating all genome-wide pairwise SNP-SNP correlation coefficients (r^2^) and assuming a 0.2 threshold. Principal Components Analysis (PCA) was performed to visualise genetic variation and possible clustering patterns using the eigenvector method implemented in eigensoft 3.0 [32,33]. For comparison, a sample of 120 domestic pigs from six breeds was used (Large White, Landrace, Duroc, Pietrain, British Saddleback and Tamworth, *n* = 20 per breed, Additional files 3 and 4). We used structure[34] for population assignment analysis with 10 runs per number of clusters (K) for K = 1-10 with 500,000 iterations and a burnin of 800,000. Optimal partitioning was evaluated using the method proposed by Evanno *et al.*[35]. Phylogenetic network analysis was performed using SplitsTree4 [36]. A number of R packages were used: Adegenet [37] for heterozygosity calculations, Hierfstat [38] for calculation of *F*_ST _values, SNPRelate [39] for the Maximum Likelihood Estimation calculation of kinship coefficients [40] based on the method of Thompson [41], and finally Vegan [42] for mantel tests in the Isolation By Distance (IBD) analysis, where genetic distance was calculated as *F*_ST_/(1- *F*_ST_) between all sampled locations.

## Results

The 351 genotyped SNPs had an overall call rate of 0.98 and 8 out of the 61075 possible pairwise SNP combinations (0.013%) interfered with linkage equilibrium. These pairwise LD SNP combinations were separated by 74-753 kb. As 50 kb is the maximum range of physical linkage in wild boar [27], the LD identified here must be caused by alternative mechanisms.

**Figure 1:**
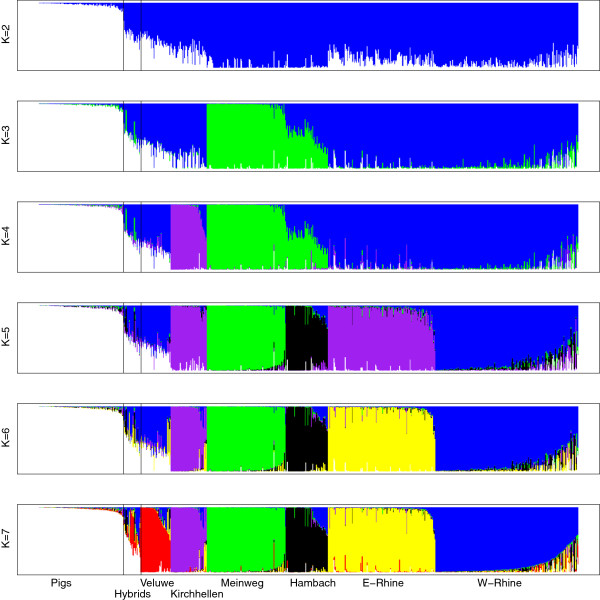
**Population assignment proportions per individual based on results from structurefor K = 2-7.** Recent wild x domestic hybrids, sampled in the field as wild boar, are delimited by vertical lines. Results for K = 5 were not ambiguous across runs. Majority rule results (*n* = 10) are presented here, but the inclusion of E-Rhine in Kirchhellen at K = 5 is not fully supported, as various alternative clustering patterns were also inferred. Evanno’s method favoured optimal partitioning at K = 7 (see Additional file 5).

We screened for wild boar-domestic pig hybrids by applying a structure likelihood assignment minimum threshold of 0.25 (25%) to a sample of domestic pigs (*n* = 120, see Methods). Individual assignment proportions for K = 1-7 are indicated in Figure 1. The assignment threshold of 0.25 was chosen based on the absence of false positive hybrids among the 88 previously studied samples [21] (Table 1). At this threshold, all five recent hybrids (up to fifth generation backcrosses with wild boar) identified previously by Allele Frequency Spectrum Assessment (based on introgressed alleles) [21] were correctly identified by structure, in contrast to the four advanced generation hybrids (Table 2). The structure algorithm identified a total of 25 recent hybrids in 645 wild boar samples (3.9%, 95% Wilson Score CI: 2.6-5.7%). This percentage is similar to previous reports [18], but here it represents recent hybrids identified by allele frequency signatures that rapidly degrade over generations, whereas previous studies may have reported hybrids based on long-term genetic signatures (e.g., mitochondrial DNA haplotypes).

**Table 1:** **Results of hybrid detection using structureat different assignment thresholds**

**Assign threshold**	**>0.30**	**>0.25**	**>0.20**	**>0.15**	**>0.10**	
Total hybrids ^1^	18	25	30	36	45	
Shared hybrids ^2^	3	5	6	6	7	
SNP60 only ^3^	6	4	3	3	2	Type II error
structure only ^4^	0	0	1	4	4	Type I error

Comparisons were made to results from Goedbloed *et al.*[21], which identified nine hybrids from a total of 88 samples using analysis of introgressed allelic states with the SNP60 genotyping beadchip.

^1^ the total number of hybrids detected in this study by structure.

^2^ the number of hybrids from the SNP60 study that was correctly detected also by structure.

^3^ the number of hybrids from the SNP60 study that were not identified by structure (type II error).

^4^ the number of individuals that were incorrectly labelled as hybrids by structure (type I error).

**Table 2:** **Detection of the nine previously studied SNP60 hybrid individuals at a structure assignment threshold of 0.25 (see Table**1**)**

	**Individual**	**Level**	**Type**
Detected	7	1^st^	Recent
	2	2^nd^	Recent
	5	3^rd^	Recent
	1	4^th^	Recent
	3	2^nd^	Recent
Not detected	9	3^rd^	Advanced
(type II error)	6	2^nd^	Advanced
	8	2^nd^	Advanced
	4	5^th^	Advanced

Individual numbering corresponds to Goedbloed *et al. *[21]. The level of introgression is based on the number of introgressed domestic alleles per individual and expressed as being equivalent to the number of generations since hybridization according to simulations [21]. The type of hybrid (recent versus advanced generation) is distinguished based on the genomic distribution of introgressed alleles (clustered or spread out respectively) [21].

**Figure 2:**
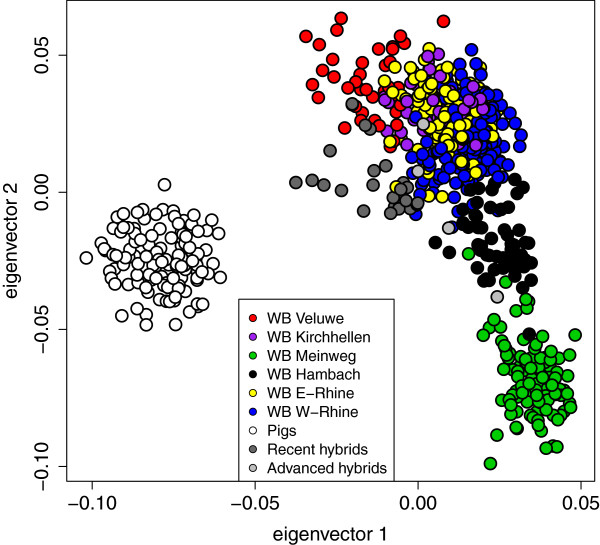
**PCA plot of the wild boar and a sample of domestic pigs, indicating genetic variation along the first two eigenvectors.** Colours correspond to Figure 1. The 25 recent wild boar x domestic pig hybrids identified by structure (threshold assignment proportion 0.25) are indicated in dark grey and four additional advanced generation hybrids with introgressed pig alleles identified in a previous study [21] are indicated in light grey.

Both structure clustering and PCA show a clear wild - domestic separation (Figures 1 and 2). The recent hybrids that are detected by structure are associated with intermediate positions between wild boar and domestic pigs as well as the origin of the plot (0,0) in the PCA (Figure 2). The four individuals identified as advanced generation hybrids using SNP60 genotyping [21] are scattered across the wild boar clusters, without visible association to the domestic pig cluster.

**Figure 3:**
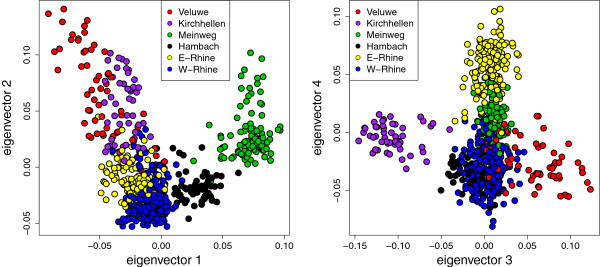
**PCA plots indicating the first four eigenvectors of the wild boar data only.** Colours indicate the six clusters identified by structure. Putative hybrids are not indicated in this figure. Eigenvectors 1–4 explain 43% of variance in the dataset.

**Figure 4:**
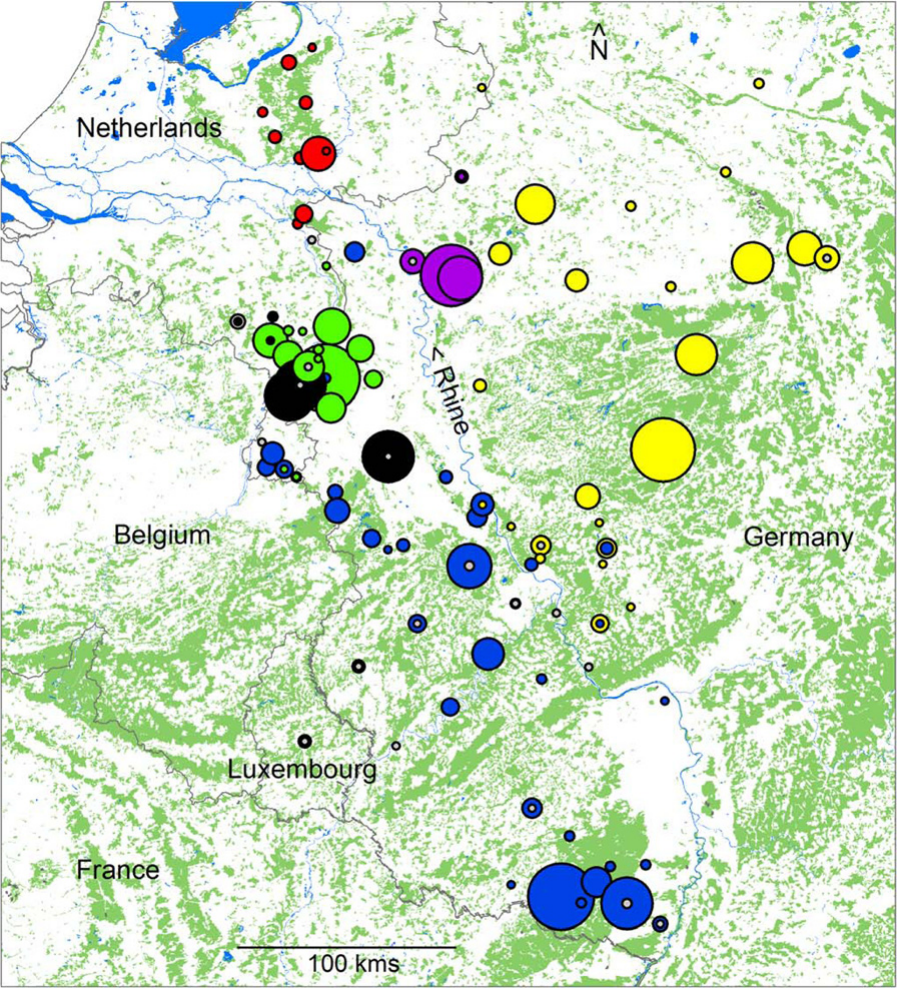
**Map of the study area indicating identified clusters.** Country borders are indicated by black lines, forests are indicated in soft green and inland water features in light blue. Dots indicate wild boar sampling sites. The size of the dot is relative to the sample size. The colours indicate genetic clustering by structure and correspond to other Figures. Hybrids identified by structure (domestic cluster assignment proportion >0.25) are indicated in grey.

Following the method of Evanno *et al. *[35], six genetic wild boar clusters were identified (Table 3, and Additional file 5). These genetic clusters were supported by separation along the first four eigenvectors in a PCA (Figure 3), which explained 43% of the total variation. *F*_ST_ values indicated moderate (0.05 < *F*_ST_ <0.15) to high (0.15 < *F*_ST_ <0.25) genetic differentiation between the inferred clusters (Table 4). In addition, the identified genetic clusters were geographically non-overlapping (Figure 4), with one possible exception (Hambach, in black). This geographic separation supports the inferred clustering and its interpretation as a biologically meaningful population structure. The River Rhine seems to act as a boundary between genetic clusters, although some gene flow occurs across the Rhine in Germany. Isolation by Distance (IBD) across clusters was near significant (*p* = 0.061), even though it was not significant within some of the clusters (Table 5). A Fisher’s combined probability test indicated that overall, the within cluster IBD is significant (*p* = 0.008) in the study area.

**Table 3:** **Genetic wild boar clusters with the corresponding sample size (*n *), observed heterozygosity (H**_**o**_**) and number of hybrids**

**Cluster**	***n***	**H**_o_^*^	**Hybrids**
Pigs	120	0.36	
Veluwe	43	0.36	0
Meinweg	112	0.35	2 (1.8%)
West Rhine	207	0.41	12 (5.8%)
Hambach	60	0.40	2 (3.3%)
East Rhine	153	0.40	3 (2.0%)
Kirchhellen	50	0.34	1 (2.0%)

The number of hybrids is based on geographic association and excludes 5 hybrids with uncertain geographic assignment.

^*^ standard errors were 0.01 or smaller.

**Table 4:** **Autosomal *F***_**ST **_**values between wild boar clusters (and domestic pigs)**

	**Kirchhellen**	**Meinweg**	**Veluwe**	**East-Rhine**	**West-Rhine**	**Hambach**
Pigs	0.193	0.234	0.150	0.158	0.162	0.192
Kirchhellen		0.215	0.170	0.125	0.124	0.171
Meinweg	0.212		0.214	0.139	0.121	0.108
Veluwe	0.149	0.189		0.111	0.108	0.165
East-Rhine	0.123	0.137	0.093		0.050	0.098
West-Rhine	0.119	0.117	0.086	0.047		0.069
Hambach	0.168	0.106	0.140	0.096	0.066	

Above the diagonal: *F*_ST_ values without hybrids. Below the diagonal: *F*_ST_ values with hybrids.

**Table 5:** Isolation by distance (IBD) analysis results for the full dataset and the different clusters separately

	**Nr locations**	**max. dist. (km)**	***p*-value**
Full dataset	101	402	0.061
Veluwe	10	76	0.326
Meinweg	15	50	0.166
Kirchhellen	4	44	0.334
Hambach	5	86	0.084
E-Rhine	30	240	0.085
W-Rhine	44	343	0.020

Results are based on mantel tests (10,000 permutations, 10 repeat average). P-values indicate the significance of IBD across sampling locations in that particular dataset or cluster. The maximum pairwise geographic distance within the cluster or dataset is also given.

**Figure 5:**
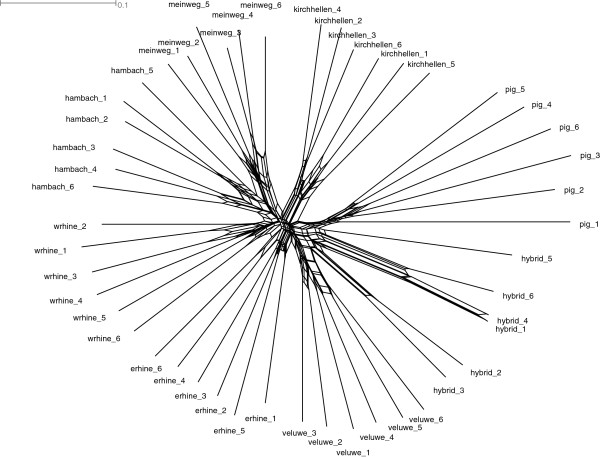
**NeighborNetwork of six representative samples per wild boar cluster and one sample per domestic pig breed.** The number of samples was chosen for optimal balance in information content and clarity of the figure. Distances are based on the uncorrected P (or Hamming) method.

Phylogenetic network analysis displayed monophyly for the domestic pigs and the six wild boar clusters (Figure 5). The hybrids identified in this study are divided into three separate lineages. We recalculated the *F*_ST_ values after excluding all identified recent hybrids to avoid possible biases due to both increased genetic variation within clusters and decreased variation across clusters caused by the scattered presence of hybrids. This exclusion of hybrids resulted in on average 0.0093 (8%) higher pairwise *F*_ST_ values (Table 4), and represents a confounding effect of scattered hybrids on population differentiation.

**Figure 6:**
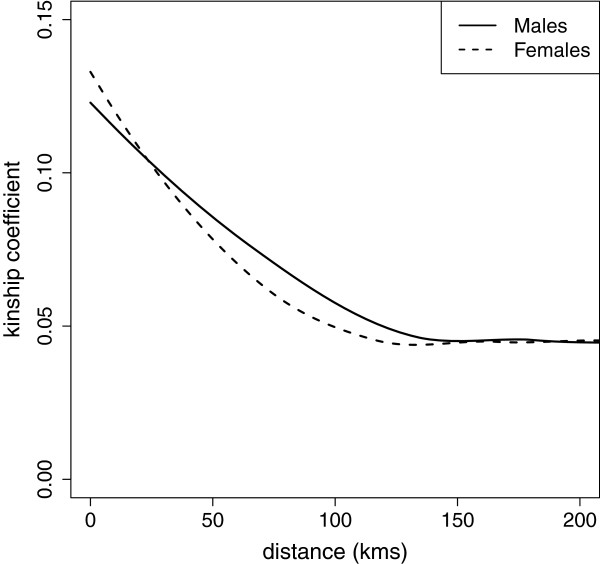
**Pairwise kinship coefficients of both sexes versus geographic distance.** Results are based on local polynomial regression analysis. Females show relative site fidelity at pairwise distances less than 25 kilometres, and males show higher kinship coefficients at distances between 25 and 150 kilometres, indicating higher dispersal rates.

The pairwise kinship coefficient is a measure of relatedness (consanguinity) between two individuals. Analysis of pairwise kinship coefficients in the wild boar dataset showed a decrease of pairwise kinship over geographic distance (Figure 6 and Additional file 6). Females displayed relative site-fidelity (higher levels of kinship at distances less than 25 km) and males showed relatively high dispersal rates (indicated by higher kinship coefficients at distances between 25 and 150 km), demonstrating effects of male-biased dispersal in this species at the population genetic level. These kinship effects of dispersal up to distances of 150 km attest to the high dispersal capacity of wild boar and correspond to occasional high dispersal distances observed in mark-recapture studies (e.g., [16]).

## Discussion

### Population genetic patterns and historical reintroductions

The largest wild boar populations in this study are found in Germany (West-Rhine and East-Rhine, Figure 4). They are relatively closely related (Table 4 and Figure 5) and most likely represent historically continuous wild boar populations. A high density of closely connected forest patches facilitates dispersal and genetic homogenisation in this part of the study area, and is only bisected by a natural barrier: the River Rhine (Figure 4). This barrier is not complete, as a few individuals seem to have crossed the Rhine in Germany. The barrier function of the River Rhine is, however, apparently sufficient to cause clear population differentiation between these clusters (*F*_ST_ =0.050, Table 4).

The wild boar found just South of the Rhine in the Netherlands, which belong to the Veluwe cluster (Figure 4), most likely represent an anthropogenic translocation event, as the intermediate terrain contains no forest and is intersected by two major rivers (the River Rhine and either the Waal or the IJssel). No wild boar were observed in this area until 1983.

The North-western section of the study area is characterised by a low level of fragmented forest cover, which is the main habitat for wild boar in Europe [9]. Historical records show that substantial forest patches appeared in this part of the study area only after the advent of artificial fertilizers and its associated reduction of landscape-wide grazing pressure at the beginning of the 20^th^ century [13]. It is unlikely that wild boar occurred in the North-western part of the study area before 1900, due to a lack of suitable habitat (forest). One cluster (Veluwe) in this North-western section certainly originates from reintroductions in 1904, and the other three clusters (Meinweg, Hambach and Kirchhellen) most likely also arose from reintroductions in the 20^th^ century. This is supported by clear genetic differentiation of each of these clusters (Table 4, Figures 1 and 5) with the other clusters, which may be explained by founder effects and subsequent reproductive isolation. The observed heterozygosity of these four populations is lower than in the Rhine populations (Table 3) supporting a historical population bottleneck or founder effect. The only exception is the Hambach cluster, which displays observed heterozygosity levels similar to the Rhine populations, but this may be explained by historical genetic introgression from domestic pigs, as discussed below. The absence of IBD in the (putatively) reintroduced populations: Veluwe, Meinweg, Hambach and Kirchhellen (Table 5), could be due to a history of introduction or translocation. On the other hand, absence of IBD may also be caused by a lack of statistical power due to small sample size (number of locations) and relatively small geographical range in these clusters. Wild boar from the Meinweg, Hambach and Kirchhellen are genetically well differentiated (Table 4, Figures 1, 3 and 5), even more so than the Veluwe cluster. The sources of the putative reintroductions in Meinweg, Hambach and Kirchhellen are unknown.

The Hambach cluster has a small geographical distribution with two localised foci (Figure 4). These two foci consist of small isolated forest patches, one of which is formed by a large brown coal mine in Germany (the Tagebau Hambach, opened in 1978, total surface 8500 hectare) and forested former refuse dump sites and fringes. This area was originally cleared of forest and only in 1980–1982 were the first dump sites (Sophienhohe) reforested, thereby creating opportunities for wild boar (re)colonisation. The other forest patch (Echt-Montfort, the Netherlands) was unoccupied by wild boar until 1983. Only one individual assigned by structure to the Hambach cluster (from the Echt-Montfort patch) was included in a previous SNP60 study [21]. This individual was then identified as an advanced generation domestic-wild hybrid. Mitochondrial DNA haplotype analysis performed in that study revealed a typical domestic pig mitochondrial haplotype in this individual. The sudden appearance of this clearly distinct wild boar cluster in Hambach and in Echt-Montfort in the 1980s, together with the evidence of genetic influences from domestic pig suggest anthropogenic introduction, most likely from a captive wild boar source. A domestic hybrid origin or influence in this cluster would also explain the relatively high levels of observed heterozygosity in such a small population (Table 3).

We assume the three populations (putatively) reintroduced in the early 20^th^ century (Veluwe, Meinweg and Kirchhellen) to have existed in complete reproductive isolation initially. However, wild boar populations across Europe have increased their numbers dramatically since the 1960s [9,14,15]. The contact zones between wild boar clusters found in this study based on the geographical overlap of clusters (e.g., Meinweg, Hambach and West-Rhine as well as Kirchhellen and East-Rhine, see Figure 4) are considered to be a consequence of these population expansions and therefore relatively recent. structure identified a relatively small number of admixed wild boar (Figure 1), all associated with contact zones. This low frequency of admixture supports a recent onset of contact between clusters.

### Identification and effects of genetic introgression from domestic pigs

The mechanism for genetic introgression from domestic pigs into wild boar populations is most likely deliberate or accidental introduction of hybrid farmed wild boar [18,21]. The structure algorithm identified 25 geographically scattered recent hybrids in 645 wild boar samples (3.9%). Hybrids are not more frequent in (putative) reintroduced populations, and seem to be recently introduced to various parts of the study area, possibly for the purpose of restocking local hunting grounds.

The structure algorithm relies solely on typical domestic pig allele frequencies for domestic-wild hybrid detection. Allele frequencies may change over time due to genetic drift and admixture with local wild boar gene pools. The figures based on hybrid detection by structure therefore only represent recent genetic introgression from domestic pigs and are likely to underestimate or disregard historical genetic introgression. Hybrid identification using a structure assignment threshold of 0.25 to the domestic pig cluster reliably identified all recent hybrids studied in a previous high-density SNP study [21], but not the advanced generation hybrids. This result indicates that allele frequency signatures from both source populations (wild and domestic) were indeed only detectable in relatively recent hybrids (approximately up to five generations of backcrossing) [21].

Phylogenetic analysis indicated multiple separate lineages within the hybrid group (Figure 5), suggesting that different hybridisation events are responsible for the detected genetic introgression from domestic pigs. This corresponds to previous findings from mtDNA haplotype analysis [21], which also suggested multiple origins of wild-domestic hybrids in this area.

If low numbers of hybrids are introduced in already occupied wild boar habitat, they would be expected to mate mostly with local wildtype individuals, leading to a rapid dilution of hybrid genetic signal over a few generations [21]. However, if hybrids are to be introduced in areas previously unoccupied by wild boar, reproduction will occur mostly among hybrids. Over time this could lead to local dominance of advanced generation hybrids and a persistent hybrid genetic signal. Advanced generation hybrids such as those produced by the latter scenario would not be identifiable as being of partly domestic origin by structure, because allele frequencies are likely to have diverged over time from those of the source populations due to genetic drift and admixture with local wild boar gene pools. However, these hybrids should be detected when analysing Allele Frequency Spectrum Assessment, which is based on introgressed allelic states (e.g., [21]). Such a scenario of older hybridisation followed by introduction to the wild and reproduction among hybrids may have shaped the Hambach cluster.

Exclusion of recent hybrids from our total dataset resulted in an average population *F*_ST_ increase of 0.0093, corresponding to 8% of the average population *F*_ST_ (Table 4). This demonstrates that domestic introgression may affect the results of population differentiation analysis in certain study systems. Here, only recent hybrids (approximately up to fifth generation backcrosses) could be excluded. Long-term effects of domestic introgression most likely also exist (e.g., in the Hambach cluster), potentially affecting genetic population structure further. The LD found in this wild boar dataset is most likely also a consequence of recent genetic introgression, although effects of population substructure and small local population sizes could not be ruled out or corrected for. As a general recommendation for population genetic analysis, we propose that hybrid detection should be performed in all cases where genetic introgression is deemed possible, to avoid associated biases in population differentiation (*F*_ST_) or LD, as well as erroneous interpretations of population structure.

## Conclusions

The presence of six well-defined genetic clusters in the study area can be attributed to two factors: the presence of a natural barrier: the River Rhine, and a history of marginalization, extinction and subsequent anthropogenic reintroductions in the Northwest of the study area. Widespread genetic signatures of recent accidental or deliberate restocking of local populations with hybrid farmed wild boar have been found, which confounded population differentiation statistics, but which do not seem to affect the existing population structure.

In this study we demonstrate the effect of past landscape and population management on current population structure in an iconic wildlife species. Effects of historical deforestation and overhunting followed by reintroductions and restocking from farms are evident. Wild boar populations in the study area are currently expanding their range. Previously isolated populations are admixing in recently formed contact zones. The relative contribution of each of the current populations to future wild boar diversity may depend on a number of factors including the effective size of populations, habitat connectivity, founder effects, restocking activity, introductions and translocations.

## Competing interests

The authors declare no competing financial or non-financial interests.

## Authors’ contributions

DJG. designed the research, performed data collection as well as laboratory and data analysis and wrote the paper. H-JM and PvH contributed to data analysis and writing of the manuscript. W.L. contributed to data collection and writing of the manuscript. RPMAC contributed to data collection and laboratory analysis. KL contributed to laboratory and data analysis. SEvW, RCY and HHTP supervised the research and contributed to the manuscript. All authors read and approved the final manuscript.

## Supplementary Material

Additional file 1 The 351 SNP genotypes of the 645 wild boar in plink format. Click here for file

Additional file 2 The 351 SNP genotypes of the 645 wild boar in plink format. Click here for file

Additional file 3 The 351 SNP genotypes of the 120 domestic pigs in plink format. Click here for file

Additional file 4 The 351 SNP genotypes of the 120 domestic pigs in plink format. Click here for file

Additional file 5 **The structure likelihood parameter L(K) ± s.d. and Evanno’s ΔK (grey line) plotted per number of clusters (K) for K = 1-10 for the wild boar dataset.** Note that the domestic pig cluster was excluded. Click here for file

Additional file 6 **Boxplot indicating the variance of kinship coefficients over 10 km geographic distance classes.** Sample sizes per distance class are given below the x-axis. Click here for file
