# Supplementary figures and images for: Reintroductions and genetic introgression from domestic pigs have shaped the genetic population structure of Northwest European wild boar
Source: BMC Genet. 2013 May 20;14:43. doi: 10.1186/1471-2156-14-43 (PMC3663677; doi:10.1186/1471-2156-14-43)

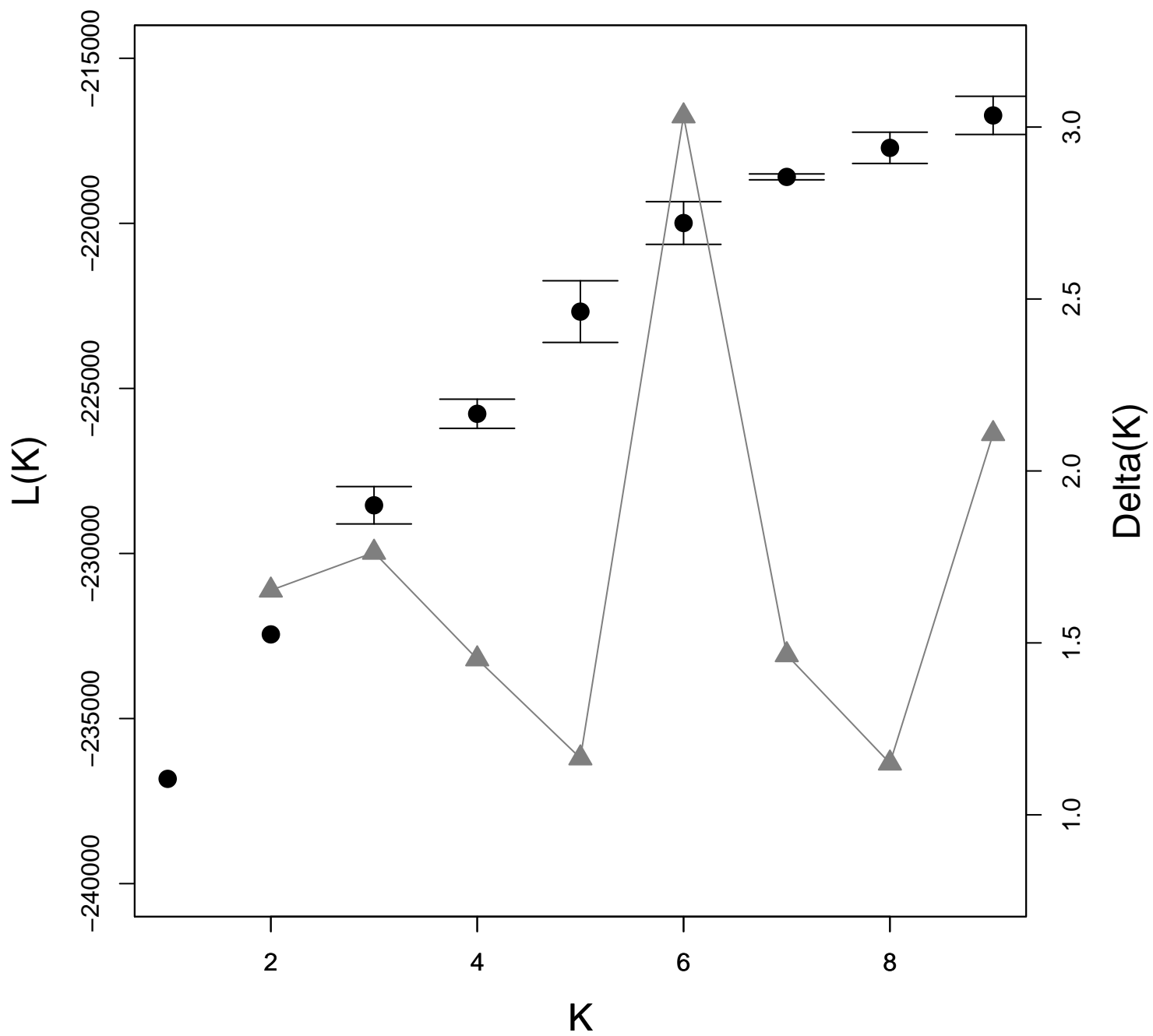

Supplement: Additional file 5 — The structure likelihood parameter L(K) ± s.d. and Evanno’s ΔK (grey line) plotted per number of clusters (K) for K = 1-10 for the wild boar dataset. Note that the domestic pig cluster was excluded. [file 1471-2156-14-43-S5.pdf]

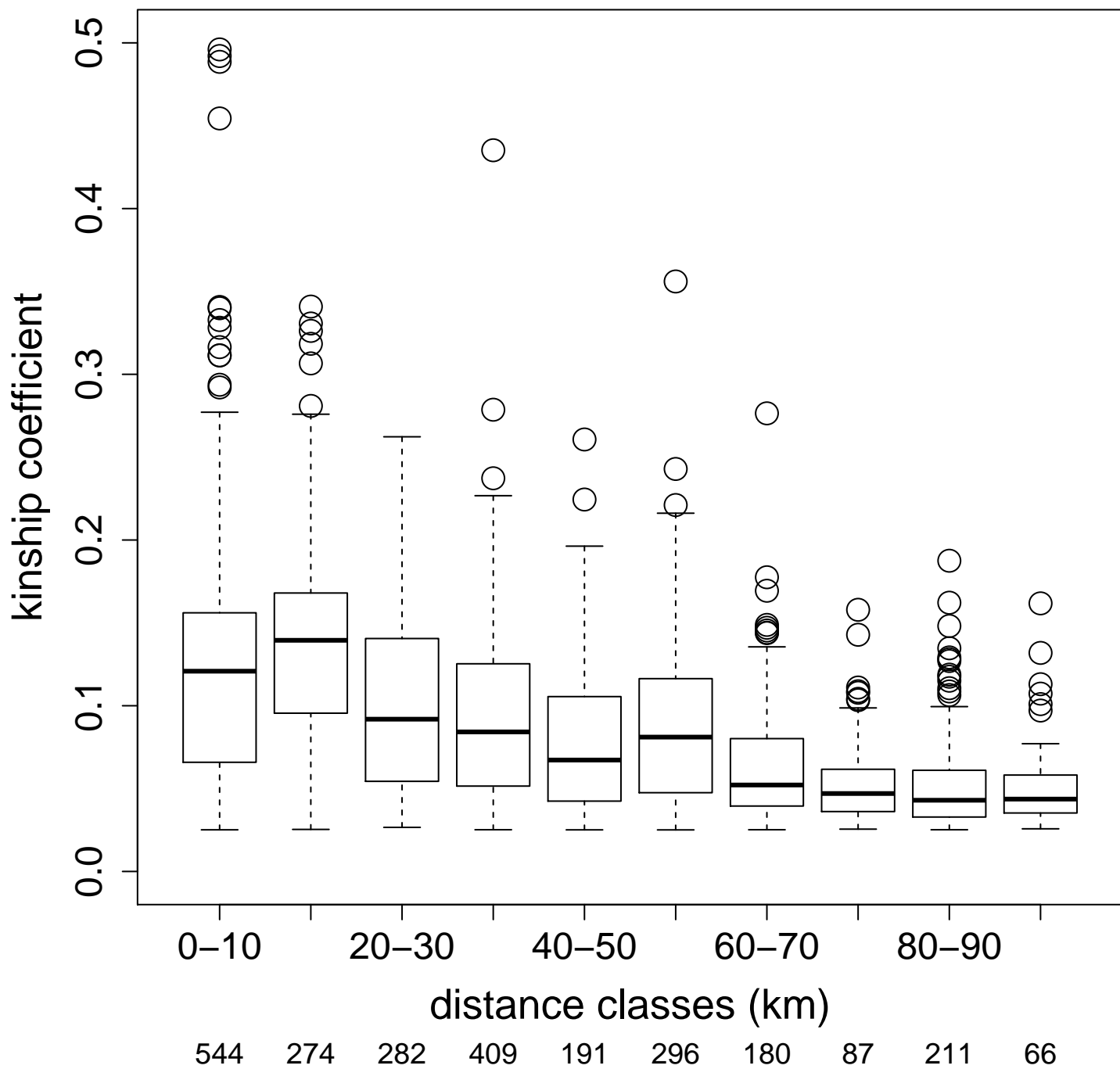

Supplement: Additional file 6 — Boxplot indicating the variance of kinship coefficients over 10 km geographic distance classes. Sample sizes per distance class are given below the x-axis. [file 1471-2156-14-43-S6.pdf]
